# The accuracy of magnetic resonance imaging diagnosis of non-osseous knee injury at Steve Biko Academic Hospital

**DOI:** 10.4102/sajr.v23i1.1754

**Published:** 2019-09-25

**Authors:** Nashil Singh, Heleen Hanekom, Farhana E. Suleman

**Affiliations:** 1 Department of Radiology, University of Pretoria, Steve Biko Academic Hospital, Pretoria, South Africa

**Keywords:** Musculoskeletal, magnetic resonance imaging, MRI, anterior cruciate ligament, ACL, posterior cruciate ligament, PCL, medial meniscus, lateral meniscus

## Abstract

**Background:**

Preoperative magnetic resonance imaging (MRI) has internationally been proven to reduce unnecessary knee arthroscopies and assist with surgical planning. This has the advantage of avoiding unnecessary surgery and the associated anaesthetic risk, as well as reducing costs. No data were found in the recently published literature assessing the accuracy of MRI interpretation of knee ligament injury in the public sector locally.

**Objectives:**

This pilot study aimed to determine the accuracy of MRI in detecting non-osseous knee injury in a resource-limited tertiary-level academic hospital in Pretoria, South Africa, compared to the gold standard arthroscopy findings.

**Method:**

This was an exploratory retrospective analysis of 39 patients who had MRI and arthroscopy at Steve Biko Academic Hospital (SBAH). True positive, true negative, false positive and false negative results were extrapolated from findings in both modalities and translated into sensitivity, specificity, positive predictive value (PPV) and negative predictive value (NPV) for each structure.

**Results:**

Negative predictive values were recorded as 97%, 81%, 90% and 100% (anterior cruciate ligament [ACL], medial meniscus [MM], lateral meniscus [LM] and posterior cruciate ligament [PCL], respectively), which were comparative to recently published international literature. The PPV results were lower than those previously evaluated at 55%, 58%, 55% and not applicable. The sensitivities and specificities of the ligaments were 83%, 58%, 83% and not applicable; and 87%, 81%, 70% and not applicable, respectively.

**Conclusion:**

Magnetic resonance imaging was found to be sensitive and specific, with a high NPV noted in all structures evaluated. Negative results can therefore be used to avoid unnecessary surgery to the benefit of the patient and state. The study reiterates that high accuracy can be obtained from MRI on a 1.5-tesla non-dedicated scanner, with interpretation by generalist radiologists.

## Introduction

The knee joint is primarily a hinge joint; however, its flexibility allows for a wide range of further movements, but at the expense of stability.^1^ This accounts for a large presenting patient population with traumatic and non-traumatic ligament injuries, including sports injuries.^2^ Internationally, the high prevalence of knee ligament derangement is widely accepted, with significant associated costs for diagnosis and treatment.^3,4^

The American College of Radiology Appropriateness Criteria suggest magnetic resonance imaging (MRI) as the primary radiological investigation for suspected non-osseous knee injury, furthermore suggesting that primary clinical examination after injury has shown a low diagnostic yield.^5,6^ The yield from the first clinical assessment alone, direct to arthroscopy, is low (between 35% and 70%)^7^ Higher accuracy from clinical assessment has been described (75%–96%) by Rayan et al., reaffirming referral to MRI following a specialist review.^8^

Magnetic resonance imaging is the gold standard imaging investigation. The modality is unparalleled in the evaluation of non-osseous knee structure derangement, being well suited for high-resolution assessment of the musculoskeletal (MSK) system, including muscle, tendon, ligament and occult bone injuries.^9,10,11^ Arthroscopy is considered the gold standard in terms of definitive diagnosis of internal derangements of the knee. It is both sensitive and specific, furthermore being both diagnostic and therapeutic.^12^ If one considers that arthroscopy is an invasive procedure that may have a negative result (4.7% reported complication rate), a major disadvantage in addition to possible patient surgical and anaesthetic complications is the expense of theatre cost and inpatient stay.^13^ A 1997 study had suggested a $680 (USD) saving of MRI before arthroscopy, and 42% of patients could have potentially avoided surgery altogether based on the MRI results.^4^ Arthroscopy is also user dependant and subject to its unique set of errors, limiting its accuracy.^14^

The effective use of preoperative MRI has been proven to reduce unnecessary surgical arthroscopies and assist with preoperative planning.^4,5,6^ This includes preoperative planning in situations including arthrofibrosis, or specific pathologies including ramp lesions or meniscal root attachment tears. These are important review areas for the radiologist and may prove to be ‘blind spots’ for the orthopaedic surgeon, depending on the arthroscopic approach used.

Recent studies comparing MRI and clinical examination to arthroscopy have shown up to 100% negative predictive value (NPV) for an anterior cruciate ligament (ACL) injury and 96% NPV for meniscal injury.^12^ A negative MRI would reduce unnecessary arthroscopies and has the added benefit of avoiding the associated costs, including theatre use, hospital stay and post-operative management. To the benefit of the patient, both surgical and anaesthetic complications may altogether be avoided.^4^

International articles have previously examined the accuracy of MRI by using arthroscopy as the gold standard. Crawford et al., in a review, examined previous literature on MRI against arthroscopy by making use of a ‘modified Coleman’ methodology to identify scientifically credible and reproducible articles on the topic. Sixty-three articles were divided into two groups; their extrapolated findings demonstrated the MRI accuracy in detecting medial meniscus (MM), ACL and posterior cruciate ligament (PCL) injuries. Sensitivities were 91%, 76% and 86%, respectively; specificities were 81%, 93% and 95%, respectively; positive predictive value (PPV) was 83%, 80% and 82%, respectively; and NPV was 90%, 91% and 96%, respectively.^7^

A study by Singla and Kansal compared ACL, PCL, MM and lateral meniscus (LM) MRI findings with those of arthroscopy. The ranges for results for the respective structures were: sensitivities of 76%–89.1%, specificities of 71.4%–94.3%, PPV of 66.7%–85.2% and NPV of 76.9%–97.1%.^15^ A prospective study by Madhusudhan et al. compared clinical examination, MRI and arthroscopy. When compared to arthroscopy, ACL imaging was 91.8% specific with a 94% NPV, whilst the corresponding results for meniscal imaging were 50% and 31%.^16^ Meniscus tears, however, showed a higher PPV of 75%.^16^ These results are however conflicting with other literature in which it was found that a definitive MRI diagnosis of a meniscal tear was made 95% of the time.^17^

The South African healthcare system is vastly different in the private and public sectors, with limitations of resources and large patient loads in the latter sector. In the public sector, this may translate into long time delays between clinical diagnosis and elective special investigations given the large population it serves. In Steve Biko Academic Hospital (SBAH), MSK imaging is performed amongst other examinations, on a non-dedicated low field 1.5-tesla (T) MRI scanner, and findings are reported by general radiologists with interest and experience; however, there is no accredited subspeciality training in MSK imaging.

Much international literature exists outlining the accuracy of MRI in diagnosis in non-osseous knee structure disruption, and the benefits to patients and hospitals alike. No local data are present, given the resource limitations at most South African tertiary state hospitals. The aim of this pilot study was to determine the accuracy (sensitivity, specificity, PPV and NPV) of MRI in detecting non-osseous knee structure injury (ACL, PCL, MM and LM) in a resource-limited tertiary-level academic hospital in Pretoria, South Africa, as compared to the arthroscopy findings.

## Methods

This was a retrospective analysis, comparing the MRI knee reports documenting non-osseous internal derangements of the knee with the corresponding knee arthroscopy report findings at SBAH, a tertiary care hospital.

Adult patients (18 years and older) who had received a knee arthroscopy (left or right knee) preceded by an MRI of the corresponding knee for the period of 01 January 2013 to 01 March 2018 were included in the study.

## Sampling method

All patients at SBAH had an MRI preceding the arthroscopy as outlined by the arthroplasty department, eliminating bias of severity of injury. A total of 39 patients were utilised, dictated by the patient records found, meeting both inclusion and exclusion criteria in the given 5-year period.

Four structures were assessed, namely ACL, PCL, MM and LM. Thereafter, the results were classified into true positive (TP), true negative (TN), false positive (FP) and false negative (FN) results. The total findings were extrapolated into sensitivity, specificity, PPV and NPV.

## Data analysis

The descriptive statistics mean, median, standard deviation and inter-quartile range were used to describe any continuous variables. Frequencies and proportions were used to describe the categorical variables, such as the presence of a tear on MRI or arthroscopy.

Sensitivity and specificity, along with positive and negative predicative values, were calculated by using arthroscopy as the gold standard. Cohen’s kappa statistics were calculated to test the agreement between MRI and arthroscopy. Tests were evaluated at the 5% level of significance. Four parameters, namely sensitivity, specificity, PPV and NPV, were calculated to assess the reliability of the MRI results.

## Ethical consideration

Ethics approval was granted by the University of Pretoria Faculty of Health Sciences Research Ethics Committee. The ethics committee was asked for waiver of patient consent as this was a retrospective study. Consent was obtained from the Chief Executive Officer of SBAH to use the reports from the hospital picture archiving and communication system (PACS) (MRI findings), as well as from the patient records (arthroscopy findings). Ethics protocol number is 442/2018.

## Results

A total of 39 patients who had arthroscopy at SBAH were evaluated. Twenty-six of the total patients were female patients and 13 patients were male patients (F:M = 2:1). The ages of the patients ranged from 18 to 69 years with a mean age of 36. Sixty-nine per cent of the patients were 40 years old or younger.

Considering arthroscopy as the gold standard, MRI findings for derangements of four structures (ACL, PCL, MM and LM) were compared to their corresponding arthroscopic theatre reports. A total of 76.9% (30 of 39) of the patients had a positive result (some structural tear) noted between the two examinations, with nine of the 39 patients having all four intact structures between both studies. True positive, TN, FP and FN results were recorded for each ligament per patient, and respective sensitivity, specificity, PPV and NPV were calculated (see Table 1).

**TABLE 1:** Results of each structure evaluated.

Structures assessed	Tear		Intact		TP	TN	FP	FN	Sensitivity (%)	Specificity (%)	PPV	NPV
	MRI	Arthroscopy	MRI	Arthroscopy								
Anterior cruciate ligament	9	6	30	33	5	29	4	1	83	87	0.55	0.97
Medial meniscus	12	12	27	27	7	24	2	3	58	81	0.58	0.81
Lateral meniscus	9	12	30	27	10	19	8	2	83	70	0.55	0.90
Posterior cruciate ligament	2	0	37	39	n/a	n/a	n/a	n/a	n/a	n/a	n/a	1.0

ACL, anterior cruciate ligament; MM, medial meniscus; LM, lateral meniscus; PCL, posterior cruciate ligament; MRI, magnetic resonance imaging; TP, true positive; TN, true negative; FP, false positive; FN, false negative; PPV, positive predictive value; NPV, negative predictive value; n/a, not applicable.

**FIGURE 1:**
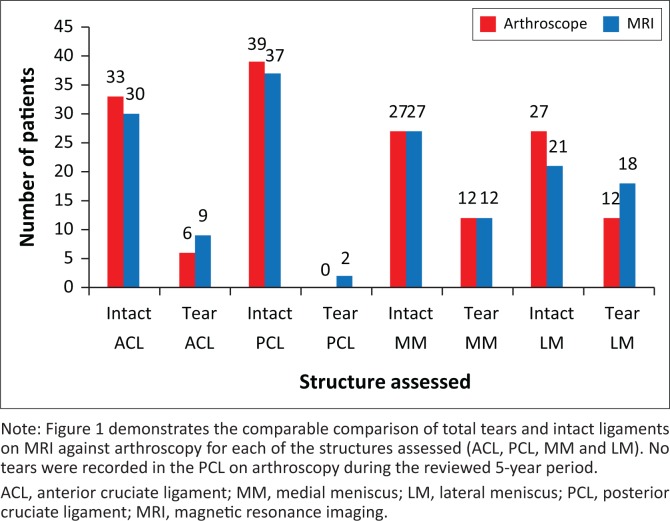
Comparison between magnetic resonance imaging and arthroscopic findings–tear versus intact ligament.

**FIGURE 2:**
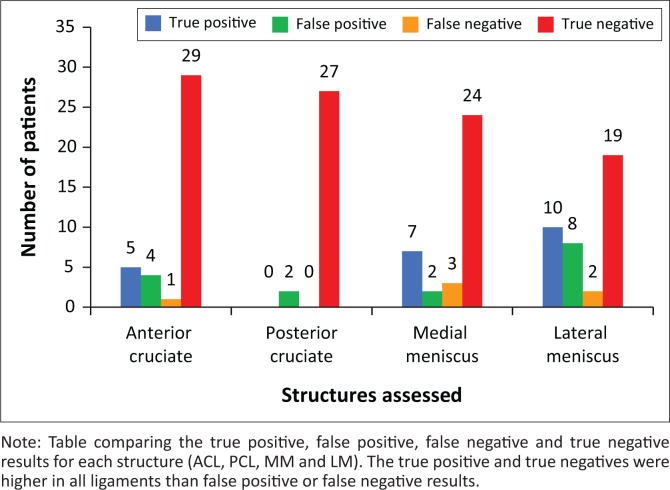
Comparison of results by ligament.

With regard to the anterior cruciate ligament, medial meniscus, lateral meniscus and posterior cruciate ligament, the findings at MRI and arthroscopy are presented in Figure 1. The resulting true positive, true negative, false positive and false negative results are presented in Figure 2. This equated to an 83%, 58%, 83%, not applicable sensitivity and 87%, 81%, 70%, not applicable specificity, respectively. A PPV of 0.55, 0.58, 0.55, not applicable and NPV of 0.97, 0.81, 0.90, 1.0 were extrapolated.

**FIGURE 3:**
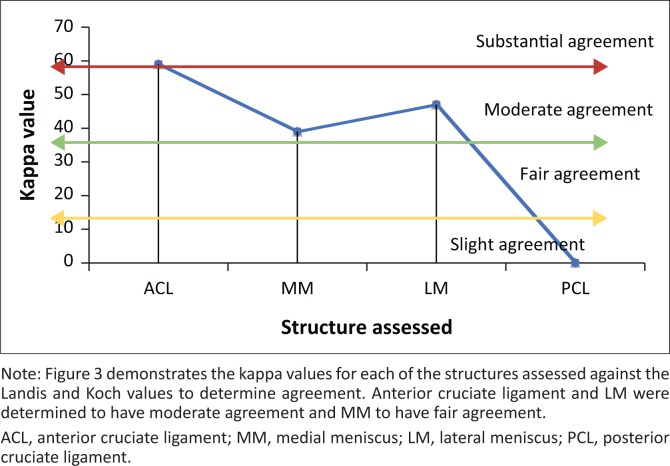
Kappa values for each structure.

Kappa testing, which accounts for the possibility of chance agreement as a more robust means to measure inter-observer agreement, was calculated for each of the ligaments. A kappa value of 0.59, 0.39 and 0.47 was found for the ACL, MM and LM, respectively. Landis and Koch regard values fair in the range of 0.21–0.40, and in moderate agreement in the range of 0.41–0.60.^18^ The PCL, however, which had two tears noted on MRI and none on arthroscopy, had a 0.00 Cohen kappa value (Figure 3).

## Discussion of results

**FIGURE 4:**
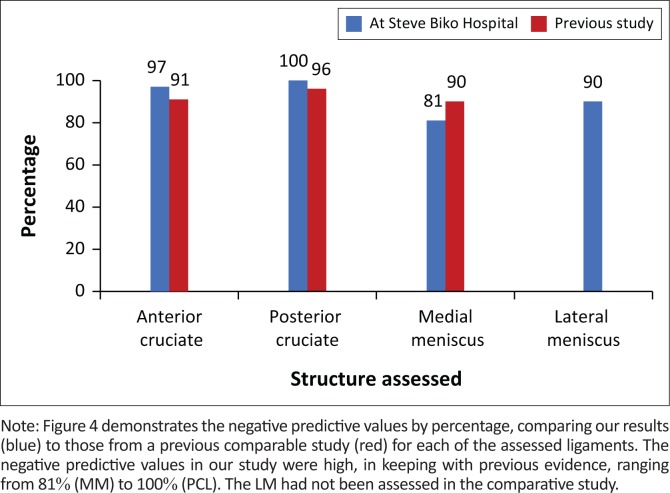
Negative predictive values.

In our study, 39 patients were managed at SBAH; the results for NPV were recorded as 97%, 81%, 90% and 100% for ACL, MM, LM and PCL, respectively. The NPV demonstrates the proportion of negative MRIs, which were TN. In our study, we found that if a patient had no tear noted on MRI, they were highly unlikely to have a tear seen on arthroscopy. This was in keeping with previous studies that demonstrated high NPV of MRI in knee ligament derangement.^7,12,15^ In one study, NPVs were recorded as 90% (MM), 91% (ACL) and 96% (PCL)^7^ (Figure 4).

The PPV is the proportion of patients with tears on MRI being true tears confirmed on arthroscopy. The PPV results were 55%, 58%, 55% and not applicable (ACL, MM, LM and PCL, respectively), which were lower than comparative previous literature. In a comparative study, the PPV for the four structures was between 66% and 85%.^15^

The sensitivities and specificities of the ligaments were 83%, 58%, 83% and not applicable and 87%, 81%, 70% and not applicable (ACL, MM, LM and PCL, respectively) Sensitivity (TP rate) is the measure of how MRI correctly identifies a tear as such. Specificity (TN rate) is the ability to exclude injury, by using the arthroscopy results as the gold standard. The sensitivities and specificities of the ligaments were comparable to previous evidence. One study, which extrapolated the results for 63 former similar credible international studies looked at MRI against arthroscopy, by using a ‘modified Coleman method’ found the sensitivities to be 76%, 91% and 86% and the specificities to be 93%, 81% and 95% for MM, ACL and PCL, respectively.^7^

**FIGURE 5:**
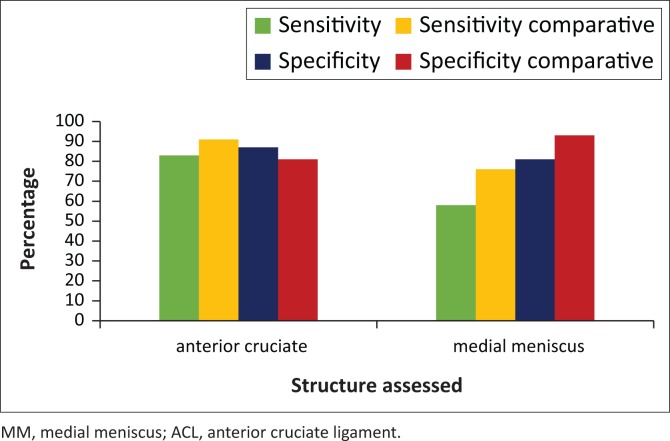
Anterior cruciate ligament and medial meniscus sensitivity and specificity compared to comparative study.

Figure 5 demonstrates the sensitivity and specificity of the ACL and MM against figures from a comparative study. Our results were corresponding to previous evidence, with the largest discordancy our low sensitivity in the MM (58%).

**FIGURE 6:**
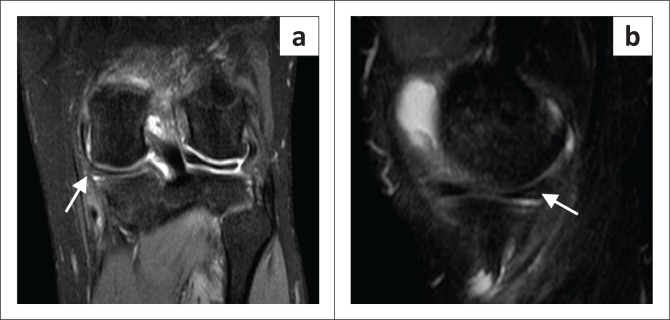
True positive medial meniscus tear: (a) coronal view and (b) sagittal view.

**FIGURE 7:**
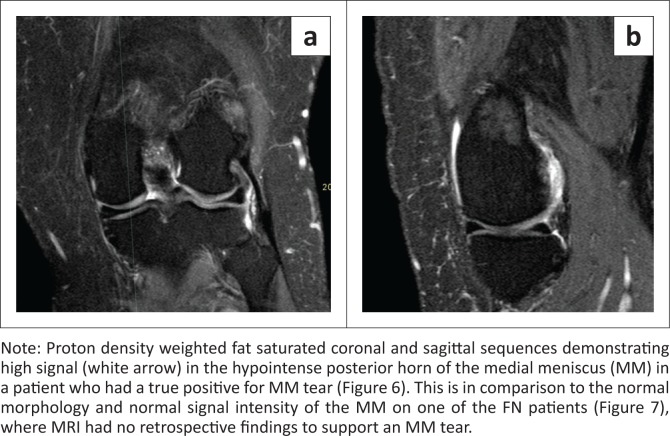
False negative medial meniscus tear: (a) coronal view and (b) sagittal view.

**FIGURE 8:**
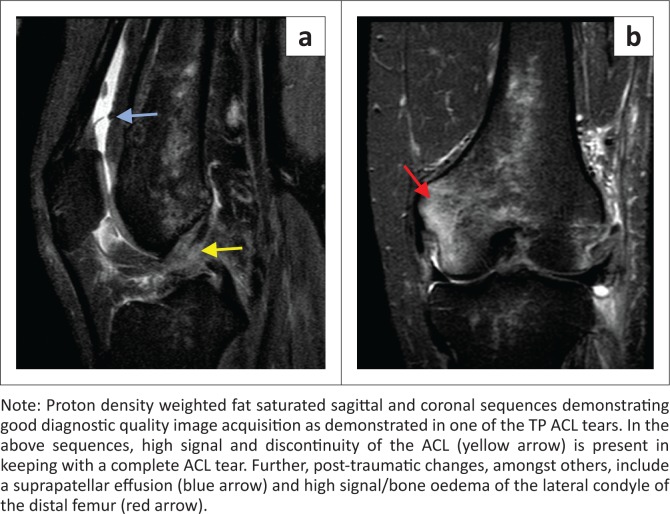
Images from a true positive anterior cruciate ligament tear: (a) sagittal view and (b) coronal view.

The largest discrepancy was noted to be the low sensitivity of the MM (58%). This stems from a high FN rate. In our study, there were three FN results (7.69%). On retrospective review of these three MRI studies, none of the classic radiological findings (high signal in contact with the superior or inferior aspect of the meniscus, nor distortion of the normal meniscus shape) were present to suggest a radiologically missed tear^17,19^ (see Figures 6 and 7). Given that the image acquisition quality was acceptable for the other structures, as supported by their higher yield correlation (Figure 8), a postulation may be that there were errors in diagnosis at arthroscopy itself, as suggested by a previous article.^20^

Unlike the MR images and reports that are stored on picture archiving and communication system (PACS) and can be retrospectively interrogated, a reviewer is purely reliant on a single hard copy of the surgical report with intraoperative pictures, which is kept in the patient’s file. The report is influenced by the skills of the performing surgeon and cannot thereafter be assessed.

Another notable difference in our study, compared to previous literature, was the time interval between MRI and arthroscopy. As the interval lengthens, there is an increased chance for discrepancy between the examination, including healing, worsening of injury or even an interim new injury occurring. A similar situation has been previously described, where healing was postulated during the long interval between the two investigations, leading to likely inconsistency in results between the examinations.^20^

**FIGURE 9:**
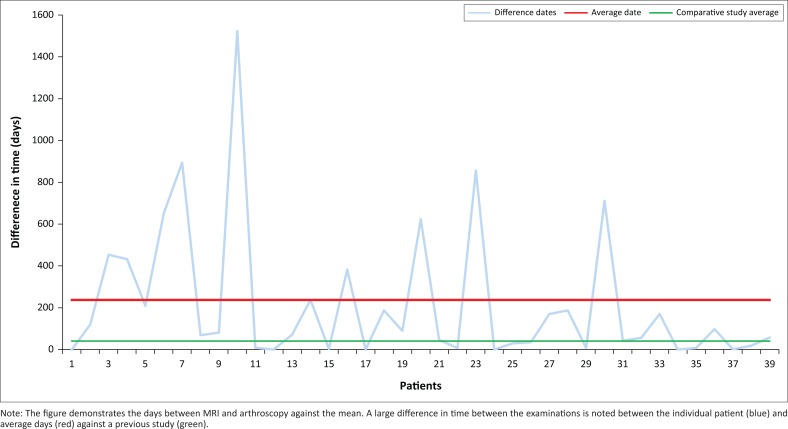
Time difference between magnetic resonance imaging and arthroscopy.

In our study, the difference in time between the two investigations ranged from 2 to 1527 days (mean of 237 days) (Figure 9), which reflects the high patient load and burden on resources in our setting. This was a much larger interval than in a previously described study (5.8 weeks or approximately 41 days).^16^ In two of our patients with the longest interval between MRI and arthroscopy (1523 days and 853 days), one study was completely congruent; however, the other had an FP LM tear (seen on MRI and not on arthroscopy). Healing may well have taken place between examinations resulting in inaccuracy of comparison.

The FN totals were low in comparison to the FPs (1, 0, 3, 2 for ACL, PCL, MM and LM, respectively). This demonstrates a tendency of the reporting radiologists to ‘overcall’ abnormalities, rather than tears being erroneously missed completely. In two instances, FP was secondary to ungraded degenerative tears (PCL and MM). The use of the word tear, rather than degeneration, reflects misinterpretation of normal aging as pathology. The lack of uniform nomenclature used within the reporting of both modalities means that inter-observer interpretation of the report is discrepant, and readers may be unsure of the tear severity grade, for example, a degenerative tear is a normal aging finding or pathological and requires further management. Grading of the tear (grade 1, 2 or 3) is a well-known classifying system, may be a more informative solution to express the degree of injury.^21^

The MRIs had all been performed on a single 1.5-T Philips Achieve MRI scanner, which was installed at the institution on 27 February 2006. This negated any variability of hardware and acquisition quality. Previous evidence suggests no advantage in using a 3-T rather than a 1.5-T MRI scanner, nor with dedicated high field extremity MRI.^22,23,10,24^ All MRI studies were reported by generalist radiologists, with no sub-specialisation in MSK, although a previous article had suggested high accuracy in similar circumstances.^25^

## Conclusion

The pilot study performed at SBAH found the MRI accuracy in determining non-osseous knee structural derangements to be comparable to previous international literature, by using arthroscopy as the gold standard. The study reiterates that high accuracy can be obtained from MRI on a 1.5-T non-dedicated scanner, with interpretation by generalist radiologists particularly for identifying cruciate ligament injuries. Magnetic resonance imaging was found to be sensitive and specific, particularly for the ACL. A high NPV was also noted in all four structures evaluated, in keeping with previous literature. This means negative results may be used to avoid an unnecessary surgical procedure to the benefit of the patient and state, and reinforces the role of MRI in excluding injury in the setting of equivocal clinical findings.
